# Effect of lncRNA MALAT1 on the Granulosa Cell Proliferation and Pregnancy Outcome in Patients With PCOS

**DOI:** 10.3389/fendo.2022.825431

**Published:** 2022-04-27

**Authors:** Mixue Tu, Yiqing Wu, Feixia Wang, Yun Huang, Yuli Qian, Jingyi Li, Pingping Lv, Yanyun Ying, Juan Liu, Yifeng Liu, Runju Zhang, Wei Zhao, Dan Zhang

**Affiliations:** ^1^ Key Laboratory of Reproductive Genetics, Ministry of Education, Department of Reproductive Endocrinology, Women’s Hospital, Zhejiang University School of Medicine, Hangzhou, China; ^2^ Women’s Reproductive Health Research Key Laboratory of Zhejiang Province, Hangzhou, China

**Keywords:** polycystic ovary syndrome, anti-Müllerian hormone, MALAT1, long noncoding RNA, granulosa cell, pregnancy outcome

## Abstract

Follicle arrest is one of the main characteristics of polycystic ovary syndrome (PCOS), the most common endocrinological disorder in reproductive-aged women. Increasing evidence proves that high anti-Mullerian hormone (AMH) levels may play an important role in follicular development. Long noncoding RNA (lncRNA) with a length of more than 200 nt is widely involved in the directional differentiation, growth, and development of cells, whereas whether lncRNA is involved in AMH’s role in follicular development is unknown. In this study, we analyzed lncRNA expression in ovarian granulosa cells (GCs) collected from women with and without PCOS *via* high-throughput sequencing. The results showed that a total of 79 noncoding transcripts were differently expressed in GCs of PCOS patients, including upregulated lncRNA MALAT1. The upregulation of MALAT1 was further confirmed by RT-qPCR in GCs from a larger cohort of PCOS patients. Furthermore, knockdown MALAT1 can promote the proliferation of KGN cell *in vitro*. These data suggested a role for MALAT1 in the development of PCOS. Meanwhile, MALAT1 and phosphorylated SMAD 1/5 (Ser463/465) protein were upregulated in KGN cells after exogenous AMH stimulation, which identified AMH perhaps as a regulator for the expression of MALAT1. We also found that MALAT1 can predict clinical pregnancy outcome to a certain extent by ROC curve analysis (area: 0.771, *p* = 0.007, 95% CI: 0.617–0.925, sensitivity: 57.1%, specificity: 91.7%). Thus, our findings revealed a role of lncRNA MALAT1 in inhibiting granulosa cell proliferation and may be correlated with pregnancy outcome in PCOS.

## Introduction

Polycystic ovary syndrome (PCOS), the most common endocrinological disorder, is characterized by chronic oligo/anovulation, hyperandrogenism, and polycystic ovaries, and it affects 8%–13% reproductive-aged women (1, 2). In addition, women with PCOS often show other disorders, including insulin resistance (IR), obesity, type 2 diabetes, cardiovascular disease, and anxiety (3). The ovary of women with PCOS exhibit more preantral or antral follicles (2–6 mm) than normal women, leading to abnormal ovulation (4). These follicles in PCOS present with a relative lack of granulosa cells and/or degenerating granulosa cells (GCs) (4, 5), indicating abonormal GC proliferation and/or apoptosis. Accumulating evidence indicates that granulosa cells are essential in determining follicular fate by providing the oocyte with nutrients and growth regulators (6). Therefore, the abnormality of GCs might be a critical factor in the pathogenesis of PCOS.

Anti-Müllerian hormone (AMH), or Müllerian-inhibiting substance (MIS), is 2~4-fold higher in serum of women with PCOS than in healthy women (7, 8). Increased serum AMH produced by granulosa cells was thought to reflect the increased number of small antral follicles (9, 10). Functional roles of AMH in ovarian folliculogenesis were to inhibit the initial recruitment of primordium follicles and reduce follicle sensitivity to follicle-stimulating hormone (FSH) (11–13). As a member of the transforming growth factor β (TGF-β) family, AMH binds to an AMH type II receptor dimer and recruits a type I receptor dimer to form a heterotetramer complex, phosphorylating mothers against decapentaplegic (SMAD)-1/5/8 (pSMAD). Phosphorylated SMAD proteins bind to co-SMAD (i.e., SMAD4), as a transcription factor complex, aggregating in the nucleus and getting involved in the regulation of target gene expression (14–16). The complexity of AMH signal opens up a lot of possibilities for the regulation of different pathological mechanisms in PCOS.

Long noncoding RNAs (lncRNAs) are a class of transcripts (>200 nucleotides) lacking protein-coding capacity (17). LncRNAs, as functional RNA, are categorized into six groups depending on the location on the genome (18). LncRNAs have various biological processes and regulate the expression of target genes *via* epigenetics, cis regulation at enhancers, and posttranscriptional regulation of mRNA processing in the nucleus and cytoplasm (19, 20). It has been reported that lncRNAs may play a crucial role in follicular development (19–22). Furthermore, several aberrant lncRNAs are expressed in different tissues of women with PCOS, including granulosa cells, follicular fluid, and peripheral blood (23–26). Despite these investigations, it remains unclear whether lncRNA mediates the pathological mechanism of high AMH in PCOS and whether there is a potential link between lncRNA and the clinical outcomes of PCOS.

In this study, we conducted RNA sequencing analysis to identify differentially expressed genes in luteinized granulosa cells obtained from women with and without PCOS. We identified that MALAT1, a lncRNA with 7–8 kb transcript located at chromosome 11, was elevated in women with PCOS. We investigated the role of MALAT1 in the granulosa cells from PCOS patients and found the potential value of MALAT1 in predicting clinical pregnancy outcomes in PCOS.

## Materials and Methods

### Human Subjects and Sample Collection

Ovarian granulosa cells and follicular fluids were collected from 68 PCOS patients and 65 controls who underwent *in vitro* fertilization (IVF) or intracytoplasmic sperm injection (ICSI) at the Center for Reproductive Medicine, Women’s Hospital, Zhejiang University School of Medicine, between July 2016 and Oct 2018. The study was approved by the ART Ethnics Committee of the Women’s Hospital, School of Medicine, Zhejiang University, and informed consent was obtained from all participants. Diagnosis of PCOS was carried out according to the revised Rotterdam consensus. Women, who were infertile due to fallopian tube factors or male factors, with regular menstruation, normal ovarian function, no clinical or biochemical profiles of hyperandrogenism, and no systemic or other gynecological and endocrine diseases served as controls. All participants were under 40 years old.

### RNA-Sequencing

Single-end libraries were synthesized using the Ion Total RNA-Seq Kit according to the supplied protocol. Library construction and sequencing was performed by Ion Proton™ Sequencer at NovelBio Bio-Pharm Technology Co. Ltd. (Shanghai, China). High-quality reads that passed the ion proton quality filters were kept for sequence analysis.

### Culture of KGN Cell Lines and AMH Treatment

The human granulosa-like KGN tumor cell line, purchased from Beina Biotechnology Co. Ltd. (Beijing, China), was grown in DMEM/F12 medium (HyClone) supplemented with 10% fetal bovine serum (BI, Beit-Haemek, Israel). The cell incubator was set at 37°C and 5% CO_2_. KGN cells were grown in 12-well plates and incubated with different doses of AMH (0, 5, 20, and 50 ng/ml) (R&D Systems, Abingdon, UK) for 24 h or within 2 h prior to RNA extraction.

### Transfection of Cells

KGN cells were seeded into 12- and 96-well plates and then transfected with three siRNAs (GenePharma, Shanghai, China) using Lipofectamine 3000 Reagent (Invitrogen, Carlsbad, CA, USA), following the supplied guidelines. After transfection, the cells were incubated for 24, 48, or 72 h before further treatments. The siRNA sequences are presented in **Supplemental Table S1**.

### RNA Isolation and RT-qPCR

Total RNA was isolated using the RNAiso Reagent and then reverse transcribed into cDNA (PrimeScript™ RT Reagent Kit). Quantitative real-time polymerase chain reaction (RT-qPCR) was performed to detect the gene expression by using the Lightcycler 480 Real-Time PCR System (Roche Diagnostics). The relative expression of RNA was performed using the △△CT method. The primer sequences of the tested genes are shown in **Supplemental Table S2**.

### Western Blotting Assay

Cultured KGN cells were treated with AMH (20 ng/ml) within 120 min and were immediately lysed in radioimmunoprecipitation assay (RIPA) lysis buffer (Beyotime Biotechnology, Shanghai, China) containing protease and phosphatase inhibitor, which were centrifuged at 13,000×*g* for 30 min at 4°C. The concentrations of protein were determined using a Pierce BCA Protein Assay Kit (Thermo). The protein denatures at 100°C for 5 min and −80°C freeze storage. Denatured proteins were separated with sodium dodecyl sulfate-polyacrylamide gel electrophoresis gels (10%) and transferred onto Hybond-NC (0.22 µm) membrane (Beijing Solarbio Science & Technology Co. Ltd., Beijing, China). Followed by blocking with 5% BSA for 1 h at room temperature, the membranes were washed with PBS (containing 0.5 ml/L Tween-20) for three times (5 min each time). Membranes were then incubated with specific primary antibodies overnight at 4°C. The antibodies used in the Western blot analysis are summarized in **Supplementary Table S3**. Next day, the membranes were washed thrice and then incubated with horseradish peroxidase (HRP)-conjugated secondary antibodies for 1 h at room temperature. ECL detection kit was used to detect the protein bands which were analyzed by ChemiDoc XRS System (Bio-Rad, Hercules, CA, USA). Quantitative analysis of protein bands was performed by ImageJ version 1.42.

### Cell Proliferation Assay

After siRNA transfection, cell viability was determined using the CCK-8 assay according to the manufacturer’s protocol and measured at 450 nm. Also, EdU Cell Proliferation Assay Kit (RiboBio, Guangzhou, China) was used following the manual book. Cell proliferation was analyzed under a fluorescent microscope.

### Clinical Pregnancy Criteria

Clinical pregnancy was diagnosed by ultrasonography on the 35th day after fresh embryo transfer with intrauterine cyst and/or fetal heartbeat. The pregnancy outcomes of the enrolled patients with or without PCOS were followed up, and whether there was a positive clinical pregnancy was included in the ROC curve analysis.

### Statistical Analysis

Statistical differences were calculated using the SPSS statistical software package (version 22.0) (IBM Corp., Armonk, NY, USA). Our data were presented as the mean ± standard deviation (SD). Student’s *t*-test was performed to analyze two different groups. Pearson was used for correlation analysis. Receiver operating characteristic curve (ROC) was used to evaluate the predictive value of the expression level of MALAT1 on the positive rate of single live birth. *p* value less than 0.05 was considered statistically significant.

## Results

### Clinical Characteristics

A total of 133 patients were included in this study, among them 8 cases (4 control patients and 4 PCOS patients) were selected for transcriptome sequencing and the others (65 control patients and 68 PCOS patients) for RT-qPCR. The major clinical characteristics of PCOS patients and the control group are shown in **Table 1**. The serum level of AMH were significantly higher in women with PCOS, which is consistent with most previous studies (9, 10).

**Table 1:** Characteristics of the PCOS and control patients.

	Cohort 1			Cohort 2			Cohort 3		
	Control (*n* = 4)	PCOS (*n* = 4)	*p*-value	Control (*n* = 13)	PCOS (*n* = 16)	*p*-value	Control (*n* = 48)	PCOS (*n* = 48)	*P*-value
Age (years)	32.00 ± 0.00	30.75 ± 0.96	0.040^*^	28.08 ± 2.81	29.56 ± 3.46	0.111	30.29 ± 3.93	28.77 ± 3.60	0.051
BMI (kg/m^2^)	22.01 ± 0.45	21.35 ± 1.33	0.31	20.52 ± 2.48	23.06 ± 2.50	0.000^***^	21.34 ± 2.55	22.03 ± 2.45	0.183
Basal FSH (IU/L)	6.91 ± 1.06	6.39 ± 0.99	0.499	6.20 ± 2.28	6.34 ± 1.59	0.422	6.56 ± 1.67	6.03 ± 2.03	0.17
Basal LH (IU/L)	5.20 ± 1.06	12.41 ± 5.99	0.055	4.87 ± 2.04	11.88± 5.21	0.000^***^	5.14 ± 2.76	10.88 ± 10.49	0.000^***^
LH/FSH	0.78 ± 0.28	1.93 ± ± 0.84	0.039^*^	0.81 ± 0.27	1.90 ± 0.88	0.000^***^	0.84 ± 0.57	1.81 ± 1.26	0.000^***^
Basal E2 (IU/L)	125.58 ± 22.61	131.50 ± 36.60	0.793	89.65 ± 40.51	115.57 ± 43.58	0.056	99.66 ± 59.73	113.37 ± 98.84	0.413
Basal PRL (ng/ml)	13.85 ± 11.87	9.025 ± 6.61	0.504	9.87 ± 9.80	5.89 ± 6.60	0.102	15.71 ± 10.34	15.48 ± 14.90	0.93
Basal P (ng/ml)	2.38 ± 0.58	1.01 ± 0.69	0.023^*^	1.17 ± 0.69	1.20 ± 1.27	0.470	1.79 ± 1.61	1.61 ± 1.47	0.475
Basal T (ng/ml)	0.98 ± 0.41	0.73 ± 0.59	0.513	0.47 ± 0.42	0.71 ± 0.70	0.140	0.72 ± 0.68	1.10 ± 1.33	0.081
AMH (ng/ml)^a^	–	–	–	3.62 ± 1.89	8.20 ± 4.14	0.000^***^	3.05 ± 2.18	9.94 ± 5.69	0.000^***^

Cohort 1 represents patients with granulosa cells for transcriptome sequencing; cohort 2 represents patients with granulosa cells for quantitative real-time PCR for differentially expressed genes; cohort 3 represents patients with granulosa cells for quantitative real-time PCR for lncRNA MALAT1. Abbreviations: BMI, body mass index; FSH, follicle-stimulating hormone; LH, basal luteinizing hormone; E2, basal estradiol; PRL, prolactin; P, progestational hormone; T, total testosterone; AMH, anti-Müllerian hormone; SEM, standard error of the mean. All results are presented as mean ± SEM. Indexes with significance differences (^*^p < 0.05; ***p < 0.001) are shown in bold. ^a^n = 22 in the control group (separately 10 in the first cohort and 12 in the second cohort) and n = 29 in the PCOS group (13 in the first cohort and 19 in the second cohort).

### Expression Profiles of Differentially Expressed Transcripts

**Figure 1:**
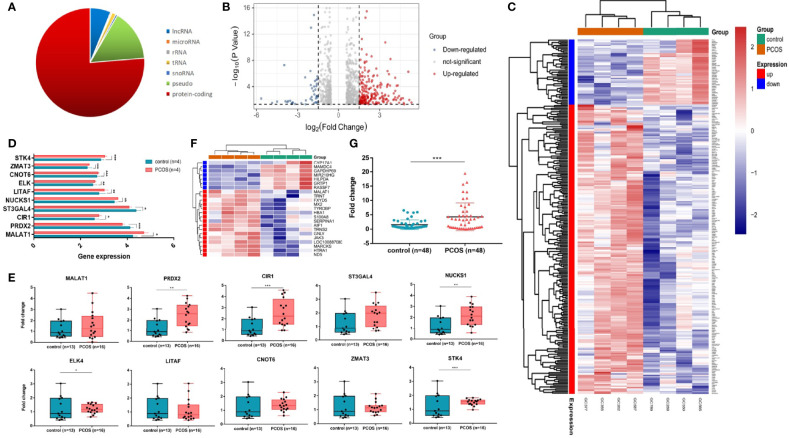
LncRNA MALAT1 is upregulated in granulosa cells of women with PCOS. **(A)** Graph showing the biotypes of RNA transcriptome sequencing profile. **(B**, **C)** Graph showing the whole differentially expressed genes. Among them, 48 were downregulated and 216 were upregulated. Red: upregulated genes; blue: downregulated genes. **(D)** Ten differentially expressed genes (DEGs) in RNA-seq. **(E)** Graph showing the DEG expression level in granulosa cells of women with or without PCOS (13 control patients and 16 PCOS patients) (*p < 0.05,**p < 0.05, ***p < 0.001). **(F)** Heatmap showing the hierarchical clustering of the most striking differentially expressed genes in patients with and without PCOS. Red: upregulated genes; green: downregulated genes. **(G)** Graph showing the expression levels of MALAT1 in a larger cohort (48 control patients and 48 PCOS patients) using RT-qPCR (***p < 0.001).

High-throughput sequencing is one way to target disease-causing genes. We evaluated the transcriptome profiling in granulosa cells of women with or without PCOS. Most differentially expressed genes were protein coding (**Figure 1A**). There were 79 noncoding genes, among them, 3 were microRNAs, 5 were snoRNAs, 2 were rRNAs, 11 were tRNAs, and 58 were long noncoding transcripts (**Figure 1A**). A total of 261 transcripts were shown to be differently expressed in GCs of PCOS patients compared with controls (log2FC > |1.5|, *p* < 0.05) (**Figures 1B, C**). Ten differently expressed genes including lncRNA and mRNA were randomly selected to verify their expression in PCOS patients of cohort 2 (13 control and 16 PCOS patients). The results showed that trends in six mRNAs (STK4, ZMAT3, CNOT6, ELK, NUCKS1, CIR1) and one lncRNA MALAT1 expression levels were consistent with the RNA-seq, while other mRNA (PRDX2, LITAF, ST3GAL4) was opposite (**Figures 1D**,**E**). In order to further target meaningful genes with high expression in GCs, we conducted further screening based on read counts (more than 50). In total, 23 genes were differentially expressed in granulosa cells of the PCOS group and control group, among which 7 genes were downregulated and 16 genes were upregulated. Three noncoding genes were found in the upregulated genes, including lncRNA MALAT1 and two tRNAs (TRNS2 and TRNT) **(** **Figure 1F**). To further verify the expression of MALAT1, 48 cases of PCOS patients with matching basic data and 48 cases of normal control granulosa cells were selected as cohort 3, respectively. The RT-qPCR results showed that the expression of MALAT1 in PCOS patients’ granulosa cells was significantly increased (*p* < 0.01, **Figure 1G**). The whole RNA-seq data were uploaded to NCBI’s Sequence Read Archive (SRA) database (accession number: PRJNA762274).

### Knockdown MALAT1 Promoted Granulosa Cell Proliferation *In Vitro*

**Figure 2:**
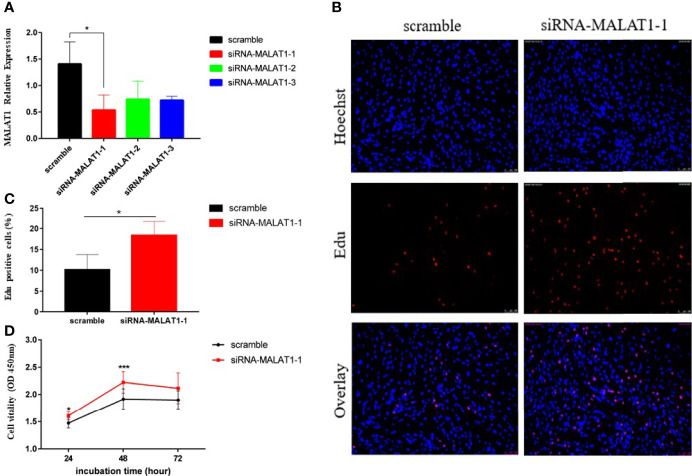
Knockdown of MALAT1 promoted KGN cell proliferation. **(A)** Graph showing the expression level of MALAT1 in KGN cells treated with three different siRNA sequences *via* RT-qPCR (^*^ *p* < 0.05). **(B)** Graphs showing the proportion of proliferation cells *via* Edu assay in granulosa cells treated with the most efficient siRNA sequence in **(A)**. The amount of cells was detected by staining with Hochest (blue), as the proliferated cells were detected by staining with EdU (red). The result was analyzed by fluorescence microscope. **(C)** Graph showing the percentages of EdU-positive cells (^*^ *p* < 0.05). **(D)** Graph showing the viability of KGN cells treated with siRNA-MALAT-1 was measured by using Cell Counting Kit-8 at the indicated time points (^*^ *p* < 0.05; ^***^ *p* < 0.01).

To investigate the role of MALAT1 in the granulosa cells of PCOS, KGN cells were transfected with three different siRNA sequences. A scramble sequence was used as control, and the knockdown efficiency of MALAT1 was verified by RT-qPCR (**Figure 2A**). We measured cell proliferation after transfection. EdU and CCK8 assays consistently showed that downregulated MALAT1 increased the proportion of proliferating cells in total cells (**Figures 2B, C**) and promoted cell proliferation ability (**Figure 2D**). These findings suggest that upregulated MALAT1 may inhibit granulosa cell proliferation.

### AMH May Upregulate MALAT1 Expression Through the SMAD Signal Pathway

**Figure 3:**
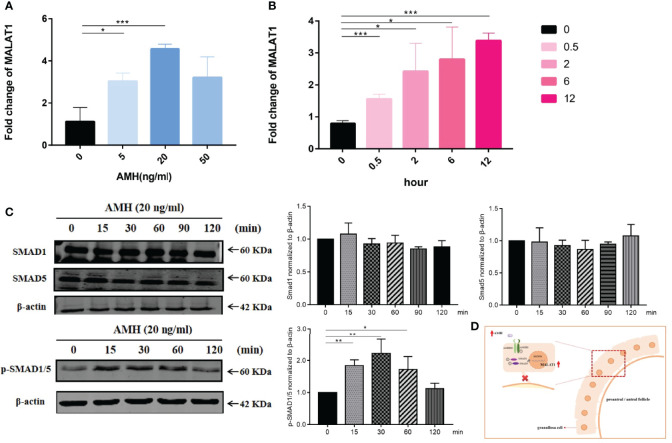
AMH upregulated SMAD signal pathway and MALAT1 expression. **(A)** KGN cells were treated with a range of AMH dose (5 to 50 ng/ml) for 24 h. The expression level of MALAT1 was determined by RT-qPCR (^*^ *p* < 0.05; ^***^ *p* < 0.01). **(B)** Graph showing the expression level of MALAT was gradually increased in KGN cells treated with 20 ng/ml AMH and was determined by RT-qPCR (^*^ *p* < 0.05; ^***^ *p* < 0.01). **(C)** Representative Western blot images using anti-pSMAD 1/5, anti-SMAD1, and anti-SMAD5 antibodies on total protein lysates extracted from KGN cells treated with AMH (20 ng/ml) for 120 minutes. Quantitative analysis of protein bands was performed by ImageJ. ^*^ *p* < 0.05; ^**^ *p* < 0.01. **(D)** Scheme graph of MALAT-induced proliferation decreased in granulosa cells. AMH upregulated the expression of MALAT *via* activing phosphorylated SMAD1/5, leading to suppress granulosa cell.

There is a lot of consensus that upregulation of AMH plays a critical role in the follicular development of PCOS. We further investigated whether AMH affected granulosa cell proliferation through the high expression of MALAT1. We first determined the effective concentration of AMH on KGN cells. We found that AMH at 20 ng/ml significantly upregulated MALAT1 after stimulating KGN cells for 24 h (**Figure 3A**). As expected, the expression of MALAT1 in KGN cells increased gradually by prolonging AMH (20 ng/ml) treatment time (**Figure 3B**), which identified AMH perhaps as regulators for the expression of MALAT1. Furthermore, we detected that AMH treatment in KGN cells increased p-SMAD1/5 levels but not total SMAD1 and SMAD5 (**Figures 3C, D**). Taken together, these data suggested that exogenous AMH increased lncRNA MALAT1 level through increasing phosphorylated SMAD1/5 protein level.

### Correlations Between MALAT1 Expression and Clinical Parameters

**Figure 4:**
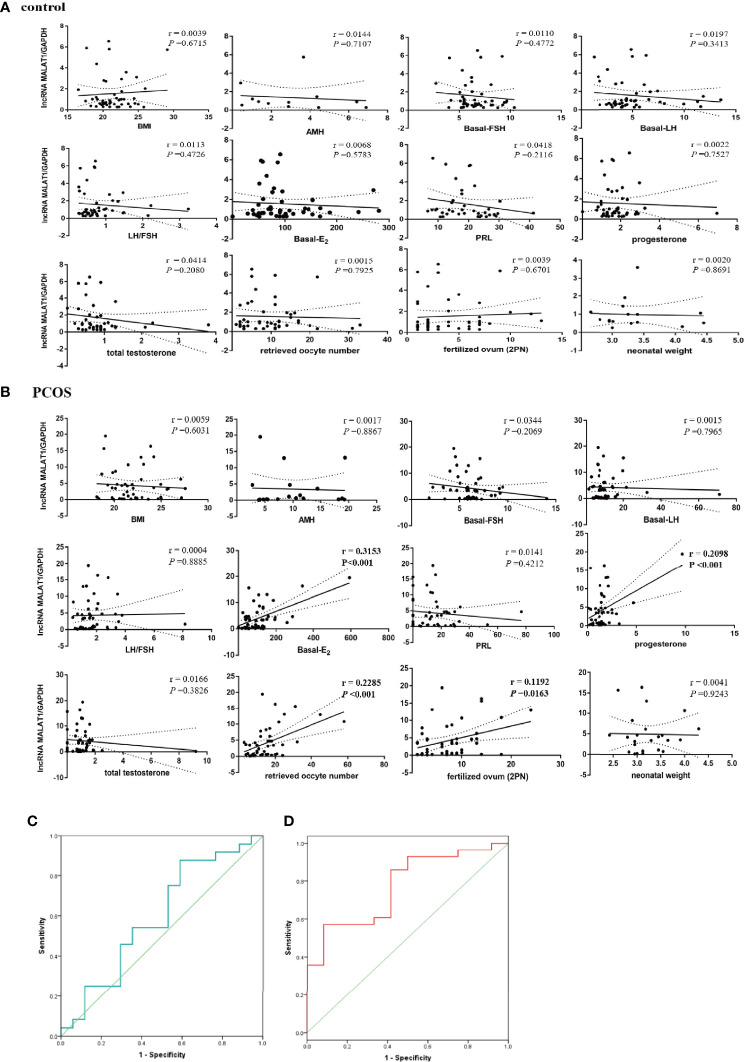
Correlations between MALAT1 expression and clinical parameters. **(A)** Graph showing correlation between the relative expression of MALAT1 in GCs of control patients and clinical parameters. **(B)** Graph showing correlation between the relative expression of MALAT1 in GCs of OCOS patients and clinical parameters. **(C)** ROC curve showing diagnosis value of MALAT in GCs for the positive clinical pregnancy in women without PCOS. **(D)** ROC curve showing diagnosis value of MALAT in GCs for the positive clinical pregnancy in women with PCOS.

Whether lncRNA MALAT1 can be used as a marker of PCOS is crucial for clinical application. We investigated the relationship between MALAT1 expression level in GCs and various clinical parameters presented in **Tables 1** and **2**. We found that high expression level of MALAT1 was positively related with basal estrogen, progesterone, retrieved oocyte number, and fertilized ovum (2PN) in PCOS patients (**Figure 4B**), while not found in control patients (**Figure 4A**). No significant correlation was exhibited between MALAT1 expression and others (BMI, basel FSH, basel LH, total testosterone, PRL, and AMH). All controlled ovulation induction programs in cohort 3 are listed in **Supplementary Table S4**.

**Table 2:** Retrieved oocyte number, embryo development, and embryo transfer outcome.

Cohort 3	Retrieved oocyte number	2PN number	Embryo transfer						
			No embryo transfer	Negative clinical pregnancy	Positive clinical pregnancy				
					Ectopic pregnancy	abortion	Twins live birth	Single live birth	Singleton neonatal weight (kg)
Control (*n* = 48)	9.85 ± 7.26	4.19 ± 3.15	6	20	1	4	2	15	3.24 ± 0.41
PCOS (*n* = 48)	15.81 ± 10.24^**^	7.71 ± 5.08^***^	8	13	1	0	2	24	3.24 ± 0.48

All results are presented as mean + SEM. Indexes with significant differences (^**^p < 0.01; ^***^p < 0.001).

### Diagnostic Values of MALAT1 Expression for Positive Clinical Pregnancy in PCOS

Binary logistic regression analysis showed that the relative expression level of MALAT1 significantly affected the clinical pregnancy rate in PCOS (*p* = 0.032), not in control patients (**Table 3**). Furthermore, ROC curve analysis was used to evaluate the diagnostic value of MALAT1 expression for positive clinical pregnancy in PCOS. The area under the curve (AUC) was 0.771 with 95% confidence interval of 0.617–0.925 (*p* = 0.007), while sensitivity was 57.1% and specificity was 91.7% (**Table 3**; **Figure 4D**). The results of the ROC analysis were not significant in the control (**Table 3**; **Figure 4C**). Those findings suggested that MALAT1 may serve as a potential diagnostic marker of positive clinical pregnancy for PCOS.

**Table 3:** Predictive value of MALAT1 on positive clinical pregnancy.

Group	Binary logistics					Area under the curve						
	B	SE	Sig.	95% confidence interval		Area	SE	Sig.	95% confidence interval		Sensitivity (%)	Specificity (%)
				Lower	Upper				Lower	Upper		
Control	0.069	0.192	0.72	0.735	1.562	0.596	0.093	0.302	0.412	0.779	87.5	41.2
PCOS	0.341	0.159	**0.032**	1.030	1.922	0.771	0.078	**0.007**	0.617	0.925	57.1	91.7

B, partial regression coefficient; SE, standard error; Sig., significance.

Indexes with significance differences (p < 0.05) are shown in bold.

## Discussion

Here, we identified that lncRNA MALAT1 was elevated in granulosa cells of women with PCOS using RNA-seq and RT-qPCR. The results of this study demonstrated that, in KGN cells, high level of AMH increased MALAT1 expression and suggest that the increase was mediated by p-SMAD1/5 protein but not SMAD1 and SMAD5. Furthermore, knockdown MALAT1 promoted KGN cell proliferation. In sum, we found that AMH increased a nuclear lncRNA, inhibiting granulosa cell proliferation in PCOS, perhaps resulting in the barrier of crosstalk between granulosa cells and eggs, and eventually the stagnation of follicle development (**Figure 3D**). However, we also found that MALAT1 expression in GCs had a positive relationship with estrogen, progesterone, retrieved oocyte number, and fertilized ovum in PCOS. Furthermore, ROC analysis implied that MALAT1 may be a potential diagnostic marker of positive clinical pregnancy for PCOS.

Notably, it has been widely recognized that arresting at the small- or medium-sized antral follicle stage is a key feature of PCOS (27, 28). The core causes of follicle arrest and failure to ovulation are still unclear. Granulosa cells, one of somatic cells in follicle, play a key part in the follicle development in PCOS. Poor responsiveness of GCs to LH and higher levels of AMH produced by GCs of antral follicles are thought to impair GC function and contribute to follicle arrest (29–31). Previous studies found that different concentrations of AMH *in vitro* could decrease granulosa cell proliferation by binding to AMHRII transmembrane receptor to active intracellular SMAD signalling pathway (32, 33). However, whether the SMAD signaling pathway can affect the function of granulosa cells by regulating the expression of lncRNA has not yet been clarified.

LncRNA MALAT1, also known as NEAT2, is highly expressed in cancer and mainly associated with tumor cell proliferation, apoptosis, migration, invasion, and functions as ceRNA (34–38). Fewer are reported in reproductive disorders. Previous studies put forward that MALAT1 is reduced in granulosa cells of women with PCOS (39, 40). On the contrary, in our study, we found that the abundance of MALAT1 was higher in the granulosa cells of women with PCOS. Compared with previous studies in PCOS patients (39, 40), PCOS patients in previous studies have higher T level but not ours (**Supplementary Table S5**). However, patients we included have higher LH and AMH levels. There is no significant difference in the LH level, and no AMH values are displayed in their studies. Based on the main difference in androgen levels, we hypothesized that MALAT1 expression in GCs might be downregulated in women with PCOS with hyperandrogenism, while MALAT1 level might be up-regulated in patients without hyperandrogenism and with high AMH. To investigate the relationship between androgen and MALAT1, *in vitro*, KGN cells were treated with a range of DHEA dose for 24 h. As expected, the expression of MALAT1 in KGN cells decreased gradually with the increase of the DHEA concentration (**Supplementary Figure S1**). *In vivo*, the decreased expression of MALAT1 in the ovaries of DHEA-induced PCOS rat model (41) also suggested that androgen inhibited MALAT1 expression. These results preliminarily supported our hypothesis that MALAT1 expression levels were inconsistent in women with PCOS with or without hyperandrogenism. In our next study, it is necessary to construct a PCOS animal model with high AMH to verify the relationship between AMH and MALAT1. Nevertheless, it was worth noting that the effect of knockdown MALAT1 on KGN cell proliferation was consistent between our study and the previous one (39). Those findings indicated that PCOS is a highly heterogeneous disease, but there may be common pathological mechanisms among diverse clinical manifestations.

In our study, we discovered that MALAT1 was mainly located in the nucleus of KGN cells (**Supplementary Figure S2**) and a certain dose of AMH upregulated lncRNA MALAT1 expression *in vitro* possibly by increasing phosphorylated SMAD1/5 protein for the first time. Our data were supported by the sufficient clinical samples and RNA-sequencing results. We believed that these clues implied that upregulated MALAT1 might play a key role in the etiology of PCOS. As part of the sample data about AMH in serum is missing, we failed to find a statistically significant correlation between MALAT1 in GCs and AMH. However, we found a positive correlation between MALAT1 with E2, P, retrieved oocyte number, and fertilized ovum, implying that MALAT1 may play a vital role in embryo development and pregnancy. Especially, the positive relationship between MALAT1 and retrieved oocyte number indicated that high expression of MALAT1 in granulosa cells probably did not affect follicle response to exogenous ovulation-stimulating hormone, and it may even be an indicator of ovarian overstimulation. Sequentially, diagnostic values of MALAT1 expression for positive clinical pregnancy in PCOS are found *via* ROC analysis. Therefore, interestingly, it is worthwhile for us to further expand the sample size and investigate profoundly the role of MALAT1 in granulosa cells of women with PCOS and its relationship with the kinds of clinical findings according to different clinical phenotypes.

It should be noted that there are limitations to this study. We have to point out that we do not provide a more detailed analysis about the expression of MALAT1 in different clinical sample subtypes. Our results lack the expression of MALAT1 in serum of women with PCOS, which could be a biomarker for diagnosis. However, these problems could be solved if we further increase the amount of patients and collect the peripherial blood samples. In addition, it remains to be elucidated that whether AMH acts directly on the expression of MALAT1 and how the SMAD signaling pathway acts on the promoter site of MALAT1 to regulate its expression. Furthermore, it needs more molecular mechanism investigation to explain how MALAT1 regulates target gene expression and destroys granulosa cell functions, leading to follicle arrest and participation in follicular maturation and fertilization after controlled ovulation induction.

Notwithstanding its limitation, our findings do uncover a possible pathological connection between lncRNA MALAT1 and AMH, which allows us to gain new insights into the mechanisms of follicular development and provides a potential diagnostic marker for positive clinical pregnancy and ovarian overstimulation in PCOS.

## Data Availability Statement

The datasets presented in this study can be found in online repositories. The names of the repository/repositories and accession number(s) can be found in the article/**Supplementary Material**.

## Ethics Statement

The studies involving human participants were reviewed and approved by the ART Ethnics Committee of the Women’s Hospital, School of Medicine, Zhejiang University. The patients/participants provided their written informed consent to participate in this study.

## Author Contributions

DZ conceived and supervised the study. MT and YW designed and conducted the study, including acquisition, analysis, and interpretation of data. YH, FW, YQ, PL, YY, and JL participated in the collection of samples. RZ and WZ participated in clinical data analysis. MT and YW drafted the manuscript. JL and YL commented on and revised the drafts of the manuscript. All authors critically reviewed, edited, and approved the manuscript.

## Funding

This study was supported by grants from the National Natural Science Foundation of China (No. 81771535) and National Key Research & Developmental Program of China (No. 2018YFC1005003, No. 2021YFC2700402).

## Conflict of Interest

The authors declare that the research was conducted in the absence of any commercial or financial relationships that could be construed as a potential conflict of interest.

## Publisher’s Note

All claims expressed in this article are solely those of the authors and do not necessarily represent those of their affiliated organizations, or those of the publisher, the editors and the reviewers. Any product that may be evaluated in this article, or claim that may be made by its manufacturer, is not guaranteed or endorsed by the publisher.
