# Supplementary material for: Effect of lncRNA MALAT1 on the Granulosa Cell Proliferation and Pregnancy Outcome in Patients With PCOS
Source: Front Endocrinol (Lausanne). 2022 Apr 27;13:825431. doi: 10.3389/fendo.2022.825431 (PMC9094420; doi:10.3389/fendo.2022.825431)
Supplement: Supplementary file 1 [file DataSheet_1.doc]

**Supplementary Table S1. siRNA sequences of MALAT1**

|  | **sequences (5’ → 3’)** |
| --- | --- |
| siRNA-MALAT1-1 | GGCAUUUGCAUCUUUAAAUTT  AUUUAAAGAUGCAAAUGCCTT |
| siRNA-MALAT1-2 | CCCUCUAAAUAAGCAAUAATT  UUAUUCCUUAUUUAGAGGGTT |
| siRNA-MALAT1-3 | GAGGUGUAAAGGGAUUUAUTT  AUAAAUCCCUUUACACCUCTT |
| scramble | UUCUCCGAACGUGUCACGUTT  ACGUGACACGUUCGGAGAATT |

**Supplementary Table S2. Primer sequences and amplification conditions used for real-time PCR**

| **Target gene** | **Primer sequences (5’ → 3’)** | **Amplification condition** |
| --- | --- | --- |
| MALAT1-forward | CTTAGAGGGTGGGCTTTTGTTG | Stage 1: 95℃, 10s  Stage 2: 95℃, 15s  60°C, 1min  Number of cycles:40 |
| MALAT1-reverse | CCATCATACTGCCAGGCTGGTT |
| CIR1-forward | AAGGAGCCCCACGAGAAAAAT |
| CIR1-reverse | ACCGAACTTGCATTGATTCCAG |
| CNOT6-forward | TACCCGCAGAACTCGGAAAC |
| CNOT6-reverse | CAGCAGCCGTCTTGTTCCAT |
| ELK4-forward | AGTGGGCAGGATTGAGGGT |
| ELK4-reverse | GCCAGTTTCTCGGCTGGATT |
| LITAF-forward | ATGTCGGTTCCAGGACCTTAC |
| LITAF-reverse | TACGAAGGAGGATTCATGCCC |
| NUCKS1-forward | GGCCTGTCAGAAATAGGAAGGT |
| NUCKS1-reverse | TTTAGCTTCTCGGGGAGATGAT |
| PRDX2-forward | GAAGCTGTCGGACTACAAAGG |
| PRDX2-reverse | TCGGTGGGGCACACAAAAG |
| ST3GAL4-forward | CAGTGGCTGGCTATGAGGG |
| ST3GAL4-reverse | GGTCGAAGTGGGCAGATTCA |
| STK4-forward | CCTCCCACATTCCGAAAACCA |
| STK4-reverse | GCACTCCTGACAAATGGGTG |
| ZMAT3-forward | | CCTTACTTCAATCCCCGCTCT | | --- | |
| ZMAT3-reverse | | CTTCGCCAGCTCCAACATTAC | | --- | |
| GAPDH-forward | GGGAAACTGTGGCGTGAT |
| GAPDH-reverse | GAGTGGGTGTCGCTGTTGA |
| ACTB-forward | CTCCATCCTGGCCTCGCTGT |
| ACTB-reverse | GCTGTCACCTTCACCGTTCC |

**Supplementary Table S3. Specifications of antibodies used in western blot analysis.**

| **Antibody** | **Antibody**  **dilution** | **Supplier of primary**  **antibody** | **Secondary antibody** | **Catalogue number** |
| --- | --- | --- | --- | --- |
| phospho-SMAD 1/5 (Ser463/465) | 1:1000 | Cell Signaling Technology | Goat Anti-Rabbit | #9516 |
| SMAD 1 | 1:1000 | Cell Signaling Technology | Goat Anti-Rabbit | #9743 |
| SMAD 5 | 1:1000 | Cell Signaling Technology | Goat Anti-Rabbit | #12534 |
| Beta Actin | 1:1000 | Cell Signaling Technology | Goat Anti-Mouse | #3700 |

**Supplementary Table S4. Summary of ovulation induction programs**

| **COS** | **Control (n=48)** | **PCOS (n=48)** |
| --- | --- | --- |
| Antagonist regimen | 16 | 19 |
| GN alone | 3 | 3 |
| GnRH-a short program | 5 | 1 |
| GnRH-a long program | 16 | 17 |
| GnRH-a ultra long program | 1 | 1 |
| Improved GnRH-a long program | 1 | 3 |
| Long follicular phase protocol | 1 | 0 |
| Mini-stimulation protocol | 3 | 2 |
| Modified microstimulus program/antagonism | 2 | 0 |
| Simulated luteal phsea ovulation induction program | 0 | 2 |

**Supplementary Table S5. Comparisons between different studies about MALAT1 in PCOS**

|  |  | Zhang et al. (2019) | Li et al. (2021) | Tu et al. (unpublish) | Chen et al. (2021) |
| --- | --- | --- | --- | --- | --- |
|  | sample | Human GCs  (30 control vs. 68 PCOS) | Human GCs  (30 control vs. 76 PCOS) | Human GCs  (48 control vs. 48 PCOS) | Ovarian tissues of DHEA-induced PCOS rat model |
| lncRNA | MALAT1 | **down** | **down** | **up** | **down** |
| Characteristics of PCOS patients (compared with control) | T | **up** | **up** | ns. |  |
|  | LH | ns. | ns. | **up** |  |
|  | FSH | ns. | ns. | ns. |  |
|  | LH/FSH | / | / | **up** |  |
|  | AMH | / | / | **up** |  |
|  | BMI | **up** | ns. | ns. |  |
| Knockdown of MALAT1 in KGN cells | apoptosis | ↓ | ↑ | / |  |
|  | Cell proliferation | ↑ | ↓ | ↑ |  |
| Overexpression of MALAT1 in rat ovarian GCs | apoptosis |  |  |  | ↓ |
|  | Cell proliferation |  |  |  | ↑ |

GCs: granulosa cells; T, testosterone; LH, luteinizing hormone; FSH, follicle stimulating hormone； AMH, anti-Mullerian hormone; BMI, body mass index
