# Supplementary material for: Effect of lncRNA MALAT1 on the Granulosa Cell Proliferation and Pregnancy Outcome in Patients With PCOS
Source: Front Endocrinol (Lausanne). 2022 Apr 27;13:825431. doi: 10.3389/fendo.2022.825431 (PMC9094420; doi:10.3389/fendo.2022.825431)
Supplement: Supplementary file 2 [file DataSheet_2.pdf]

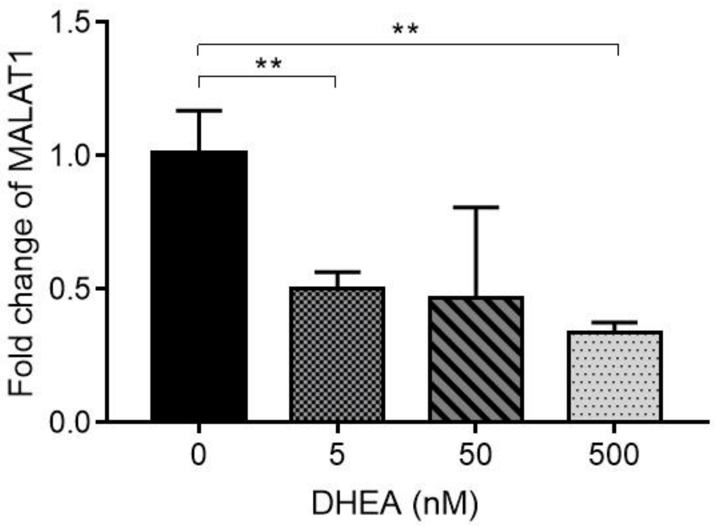

Supplementary Figure1. KGN cells were treated with a rang of DHEA does (0, 5, 50, 500 nM) for 24 hours. The expression level of MALAT1 was determined by RT-qPCR. *P<0.05, **P<0.01,***P<0.001

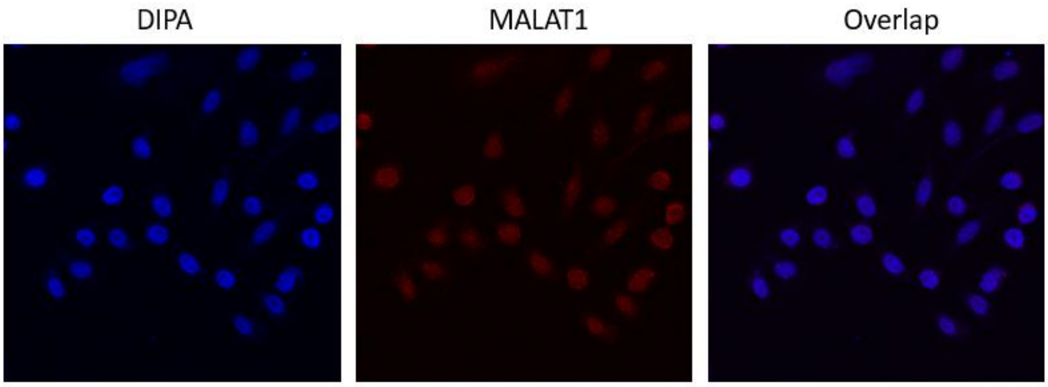

Supplementary Figure2. MALAT1 location in KGN cell was determined using the fluorescence in situ hybridization (FISH) assay according to the manufacturer's protocol (GenePharma, Shanghai, China). Malat1 probe sequence : 5'-tacttccgttacgaaagtcc-3'. The 5' end is modified by FAM. The image were analyzed by FV10-ASW 4.2 Viewer.
